# Supplementary material for: Cortical population activity within a preserved neural manifold underlies multiple motor behaviors
Source: Nat Commun. 2018 Oct 12;9:4233. doi: 10.1038/s41467-018-06560-z (PMC6185944; doi:10.1038/s41467-018-06560-z)
Supplement: Supplementary file 1 — Supplementary Information [file 41467_2018_6560_MOESM1_ESM.pdf]

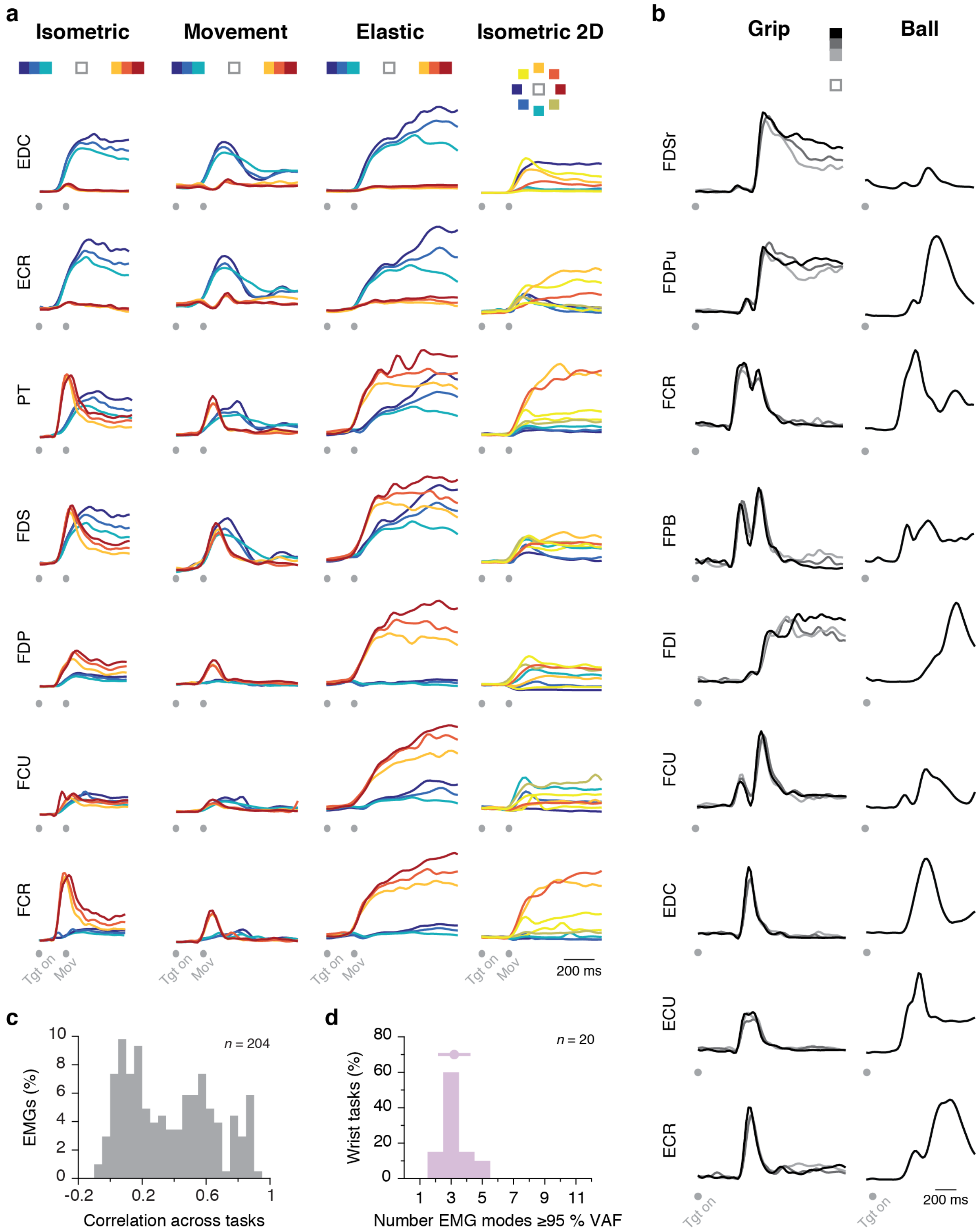

Supplementary Figure 1. Examples of muscle activity (EMGs) illustrate their broad diversity, both across tasks and across targets for a given task. **(a)** EMG envelope of seven wrist and hand muscles for one session in which Monkey J performed all four wrist tasks. EMGs are colored according to target location (top row). **(b)** EMG envelope of nine wrist and hand muscles for one session in which Monkey T performed the two reach-to-grasp tasks. Data presented as in (a). **(c)** Correlation of the activity of each muscle across two different tasks, pooled over all muscles, pairs of tasks, sessions, and monkeys. Note the strong presence of low correlation instances. **(d)** Number of EMG modes that account for ≥ 95 % of the total amount of variance for the wrist tasks. Error bar: mean ± SD. Muscles: ECR, extensor carpi radialis; ECU, extensor carpi ulnaris; FCR, flexor carpi radialis; FCU, flexor carpi ulnaris; PT, pronator teres; EDC, extensor digitorum communis; FDS, flexor digitorum superficialis (FDSr, FDS radial side); FDP, flexor digitorum profundus (FDPu, ulnar side); FPB, flexor pollicis brevis; FDI, first dorsal interosseous.

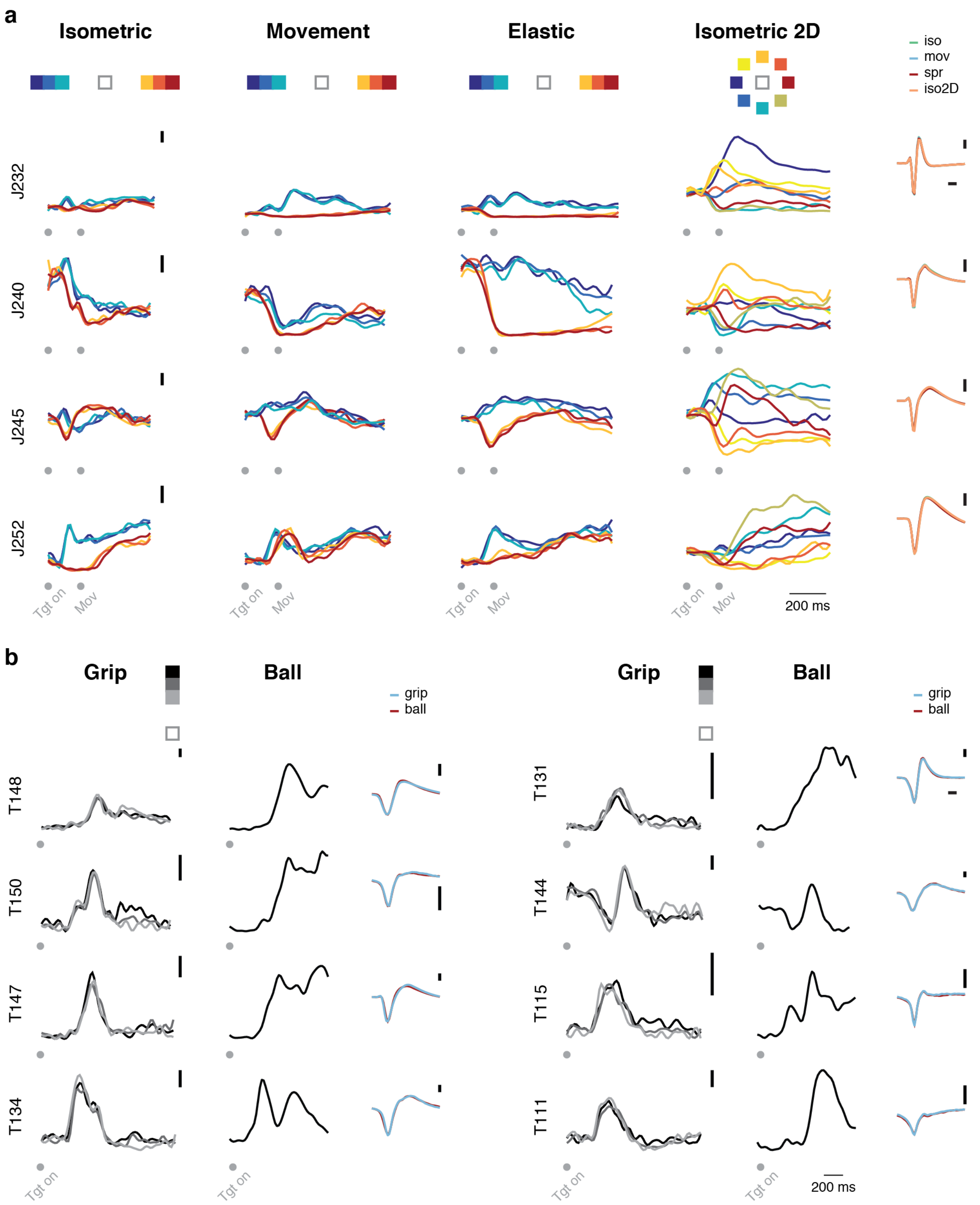

Supplementary Figure 2. Examples of neural unit activity patterns illustrate their broad diversity and their complex changes across tasks. **(a)** Averaged activity of four additional units for one session in which Monkey J performed all four wrist tasks (same session as in Fig. 2). Traces are colored according to target location (top row). Right-most columns: action potential waveform for each task; each in a different color. Note the excellent overlap. **(b)** Averaged activity of eight units for one session in which Monkey T performed the two reach-to-grasp tasks. Data presented as in (a). Scale bars: for the averaged neural activity, 10 pps; for the action potential waveforms: horizontal, 200 μs; vertical, 100 μV (a), 40 μV (b).

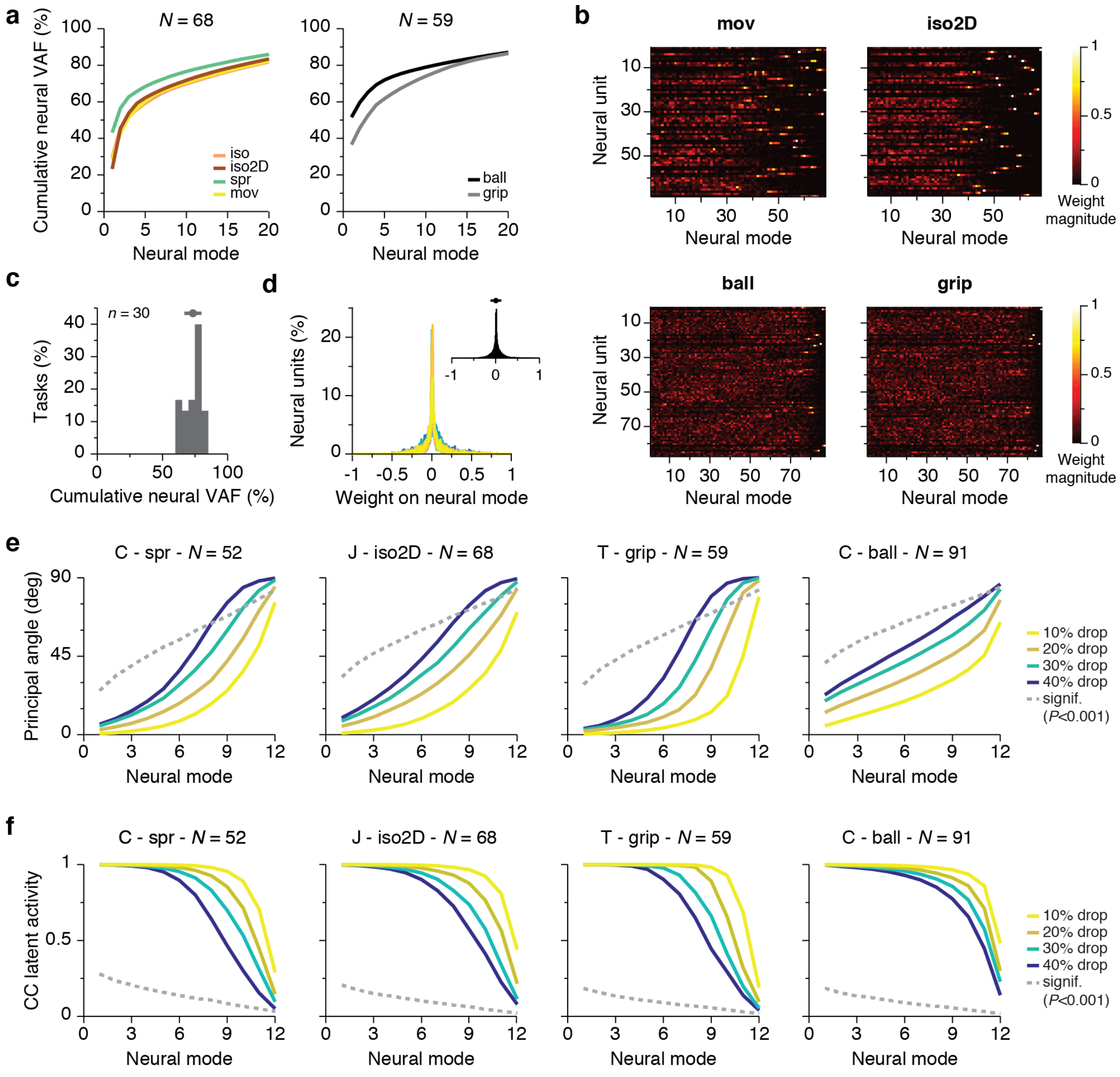

Supplementary Figure 3. The neural manifold captures population-wide activity patterns. Both the orientation of the neural manifold and the latent activity are preserved when dropping up to 40% of all neural units. **(a)** Cumulative variance accounted for (VAF) as function of manifold dimensionality for the four wrist tasks in one session of Monkey J (left), and for the two reach-to-grasp tasks in one session of Monkey T (right). *N*: number of neural units. **(b)** Absolute value of the weights of the neural units onto each PC neural mode for the movement task and 2-D isometric task in one session of Monkey J (top), and for the ball and grip tasks in one session of Monkey C (bottom). Note that the leading neural modes show participation of most units. **(c)** Distribution of VAF by a 12-D manifold, pooled over all monkeys, sessions, and tasks. Error bar: mean ± SD. **(d)** Distribution of neural unit weights onto the leading 12 neural modes. Each trace shows the weight distribution across all neural units for each task; the figure shows each task from each session and monkey in a different color. Inset: histogram summarizing all the pooled data (same units as main figure in the panel; error bar: mean ± SD). The units exhibit small weights for all the tasks, with no outliers with large weights; the leading neural modes thus do reflect population-wide activity patterns. **(e)** Principal angles between two 12-D neural manifolds identified after randomly dropping a given percentage of units. Colored traces (see legend for color code) show mean principal angle across 100 random drops; title: monkey and task; *N*: number of units. Leading principal angles were far below the $P<0.001$ significance level (dashed gray line). **(f)** Canonical correlations (CC) computed after randomly dropping a given percentage of units. Data presented as in (e).

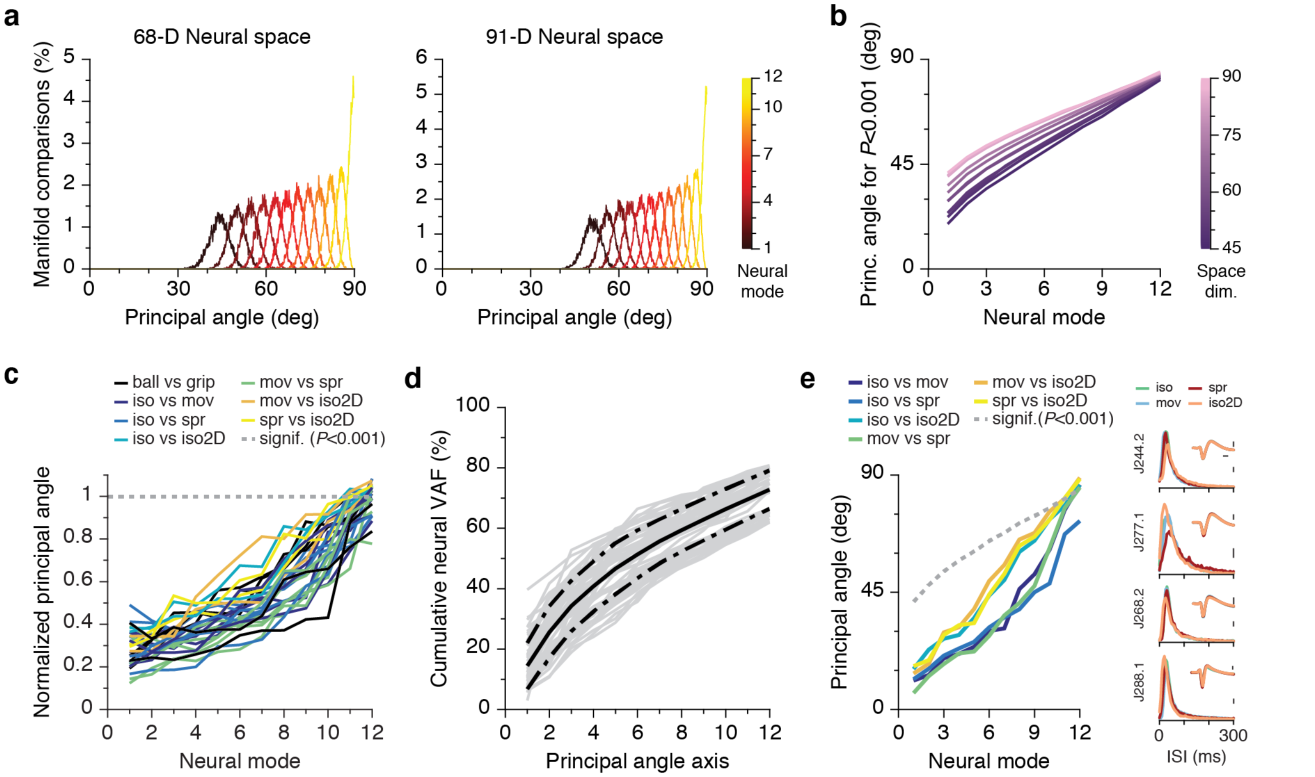

Supplementary Figure 4. Controls and additional results for the comparison of neural manifolds from different tasks using principal angles. **(a)** Example distributions for all 12 principal angles between pairs of 12-D manifolds from 10,000 surrogate datasets for one example comparison of wrist tasks (N=68; from Monkey J), and one example comparison of reach-to-grasp tasks (N=91; from Monkey C). The surrogate datasets were generated with the tensor maximum entropy method, preserving the covariance over time and the covariance across targets of the original data. **(b)** Principal angles that define the P<0.001 significance threshold between pairs of 12-D manifolds in neural spaces with dimensionality equal to each of our datasets, generated as in (a). For visualization purposes, we show the mean threshold across all task-comparisons for each dataset. Principal angles below their corresponding significance threshold indicate a large degree of similarity in manifold orientation. **(c)** Normalized principal angles between the 12-D neural manifolds for all pairs of tasks. Data were normalized by dividing the experimentally obtained principal angles by the significance threshold (P<0.001) for the surrogate principal angles between manifolds generated using tensor maximum entropy. Normalized principal angles <1 are significantly small, indicating that the structure of the neural covariation patterns was well preserved across wrist and reach-to-grasp tasks. **(d)** Neural variance accounted for (VAF) along the manifold directions identified with principal angles (gray traces: individual comparisons; black traces: mean ± s.d.). **(e)** Using single units or multi-units to compute the neural manifold yields similar principal angle results. Principal angles for the session of wrist tasks from Monkey J shown in Figure 3a; in this case the task-specific neural manifolds were computed based on single units. Each pairwise comparison is shown as one colored trace (see legend). Leading principal angles were far below the P<0.001 significance level (dashed gray line), indicating significant similarities in the structure of the neural modes across tasks. The inset (right) shows ISI distributions and average action potential waveforms (inset) for four example single units for the same dataset. Each task is shown in a different color (legend). Scale bars: ISI, 1 % probability; action potential waveforms: horizontal, 100 µs; vertical, 100 µV.

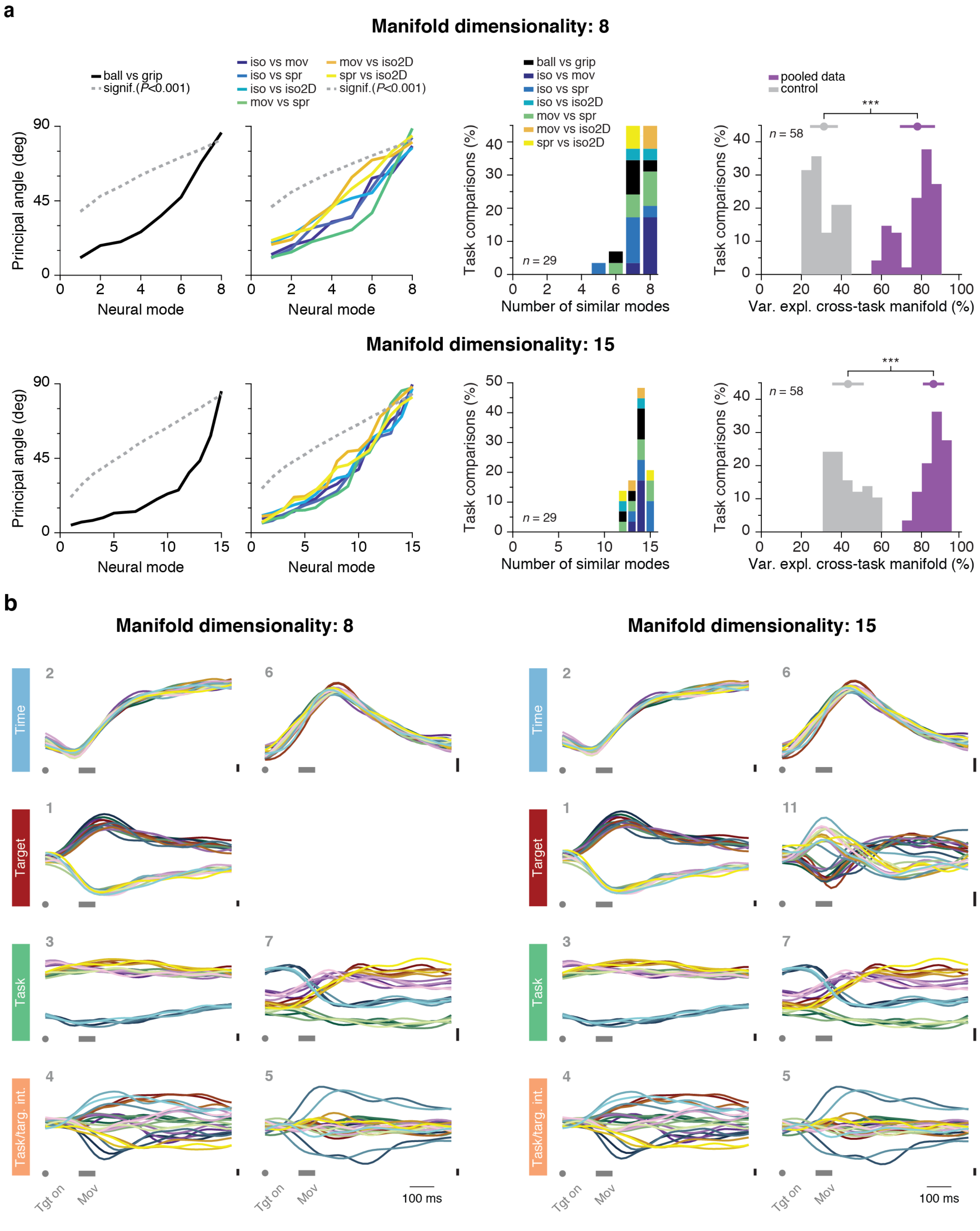

Supplementary Figure 5. The main results in the paper hold across different manifold dimensionalities. **(a)** Quantification of the similarity of the neural modes for two different tasks using principal angles. This panel replicates Figure 3 for manifold dimensionalities 8 and 15. **(b)** Task-specific and task-independent activity of dPCA modes. This panel replicates Figure 4b for manifold dimensionalities 8 and 15; see Figure 4d for legend.

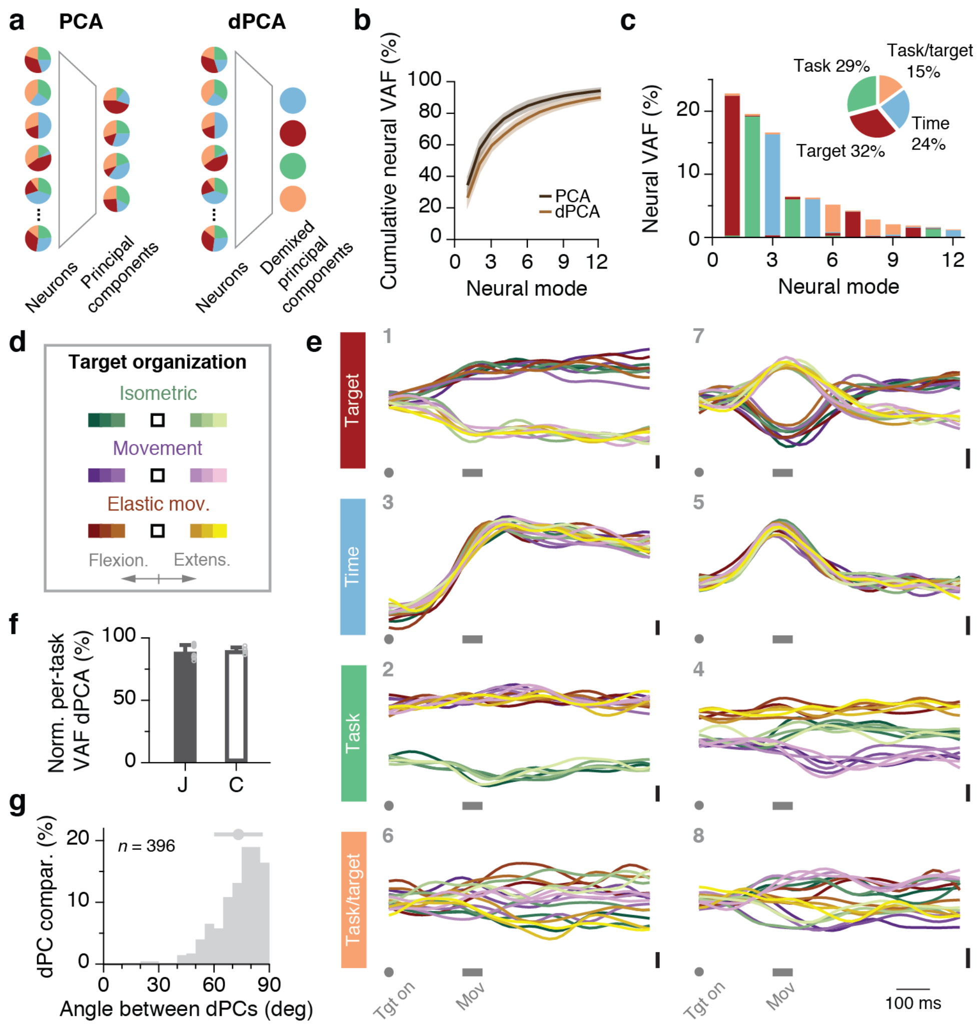

Supplementary Figure 6. Additional information on task-specific and task-independent activity for the neural modes identified by dPCA. **(a)** Dimensionality reduction with PCA and dPCA. Unlike PCA, dPCA identifies neural modes associated with relevant behavioral parameters (adapted from Ref. 51). **(b)** Cumulative neural variance accounted for (VAF) by 12-D manifolds spanning all wrist tasks over all sessions (mean ± s.d., trace and colored strip). The neural modes that spanned the manifolds correspond to either PCA or dPCA (legend). In this case, we computed the VAF using trial-averaged data, as required by dPCA. **(c)** Neural VAF by each dPC neural mode, and its relation to behavioral parameters for one example session from Monkey C. Inset: VAF associated with each behavioral parameter across all twelve modes. **(d)** Target locations for each wrist task and color code for each task/target combination for the session shown in (c). Each task is represented using a different color; extension targets are shown in dark colors and flexion targets in light colors. **(e)** Activity of eight dPC neural modes, grouped in four sets based on the behavioral parameter they are most strongly associated with. The number on the top left of each panel indicates the ranking of that neural mode in terms of VAF, as in (c). Each row corresponds to one behavioral parameter (vertical labels on the left). Each panel has 18 traces, corresponding to each of the 18 task-target combinations defined in (d). **(f)** Ratio of the VAF when projecting the task-specific neural data onto the dPCA manifold that spans all the tasks in one session, compared to the VAF when projecting the same data onto its corresponding task-specific PCA manifold. Data averaged across all wrist tasks and sessions, separately for each monkey. Bars: mean + s.d.; grey circles: individual manifold comparisons. **(g)** Distribution of pairwise angles between dPC neural modes. Most of these angles were close to orthogonal. Data pooled across all monkeys, wrist sessions, tasks, and dPC mode comparisons. Error bar: mean ± SD.

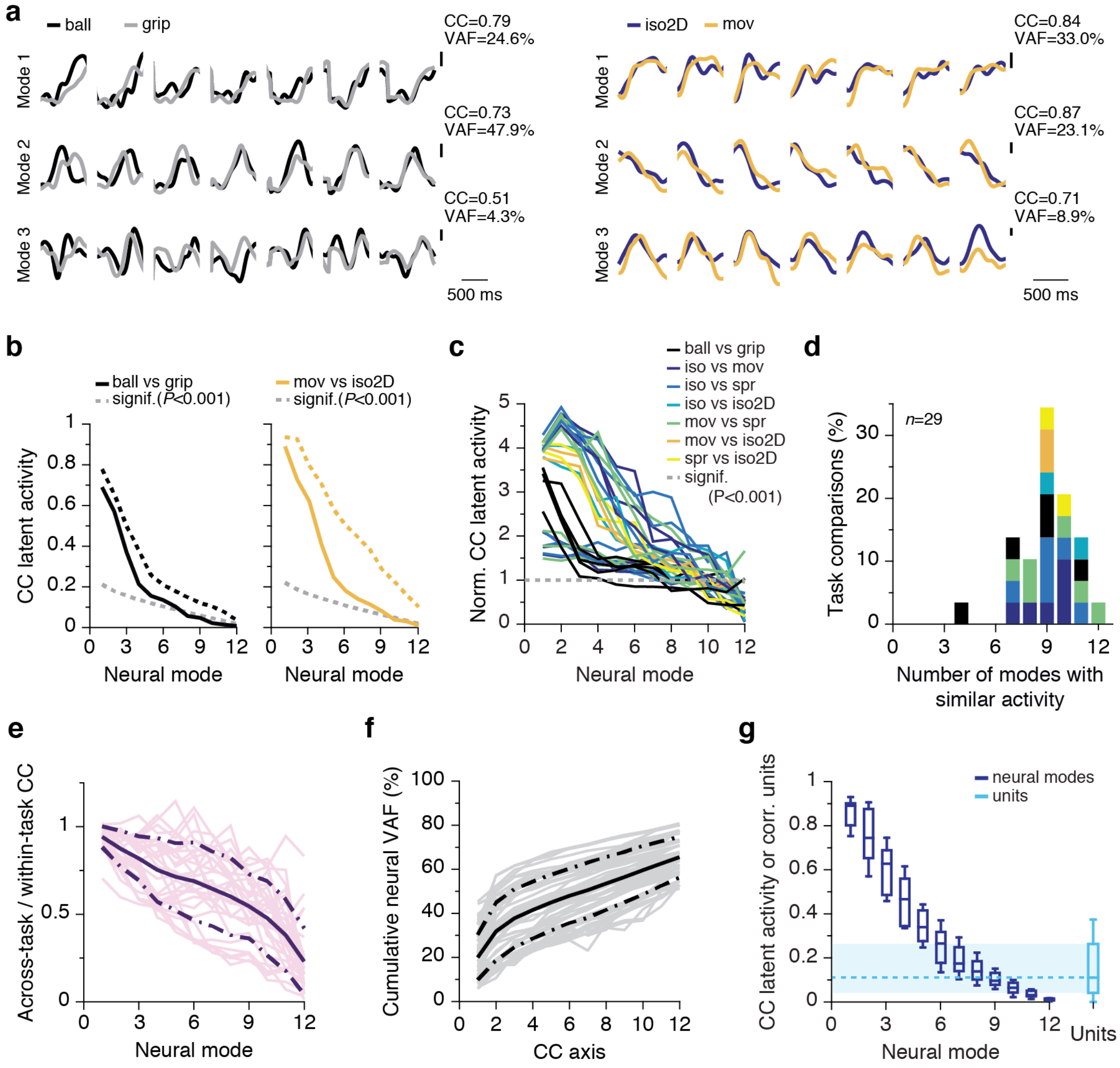

Supplementary Figure 7. Canonical correlations between two different tasks quantify the similarity of the respective latent activities. **(a)** Leading three CCs for seven trials of the ball and power grip tasks for one session from Monkey T (left), and for seven trials of 1-D movement and 2-D isometric wrist tasks for one session from Monkey J (right). Percentage of VAF along the respective direction identified by CCA, averaged across the two tasks being compared. **(b)** CC between the two reach-to-grasp (left) and the two wrist tasks (right) shown in (a). The dashed colored lines are upper bounds for across-task comparisons provided by the maximum of the two corresponding within-task CCs (same color); the significance threshold ($P<0.001$, obtained with bootstrapping) is shown as a dashed gray line. **(c)** Normalized CCs for all pairs of tasks. Data were normalized by dividing the experimentally obtained CCs by the surrogate CCs ($P<0.001$). Normalized CCs >1 are significantly large. The large value of many leading normalized CCs identifies components of the latent activity that were well-correlated across tasks. **(d)** Number of significant CCs ($P<0.001$) pooled over all sessions for all monkeys and all pairs of tasks; same color code as in (c). **(e)** Ratio of the across-task CCs to the corresponding within-task CC, as in (b). Data for all monkeys, sessions, and task comparisons (pink traces: individual comparisons; purple traces: mean ± s.d.). Ratios <1 confirm that within-task correlations provide an upper bound to across-task correlations. The leading ratios are quite large (the average ratio for the leading 6 dimensions was always ≥0.75), indicating quite high across-talk correlations over several dimensions. **(f)** Cumulative variance accounted for (VAF) along the manifold directions identified with CCA (gray traces: individual comparisons; black traces: mean ± s.d.). **(g)** Comparison of the similarity of the latent activity across two tasks, computed with CCA, and the corresponding similarity of neural unit activity, computed using pairwise correlations. Data pooled over all monkeys, sessions, and pairwise task comparisons. Boxplots show median: center line; interquartile range: box; and data range (0.5 × interquartile range): whiskers. On average, the dynamic similarity for the leading six neural modes exceeded that of the neural units.

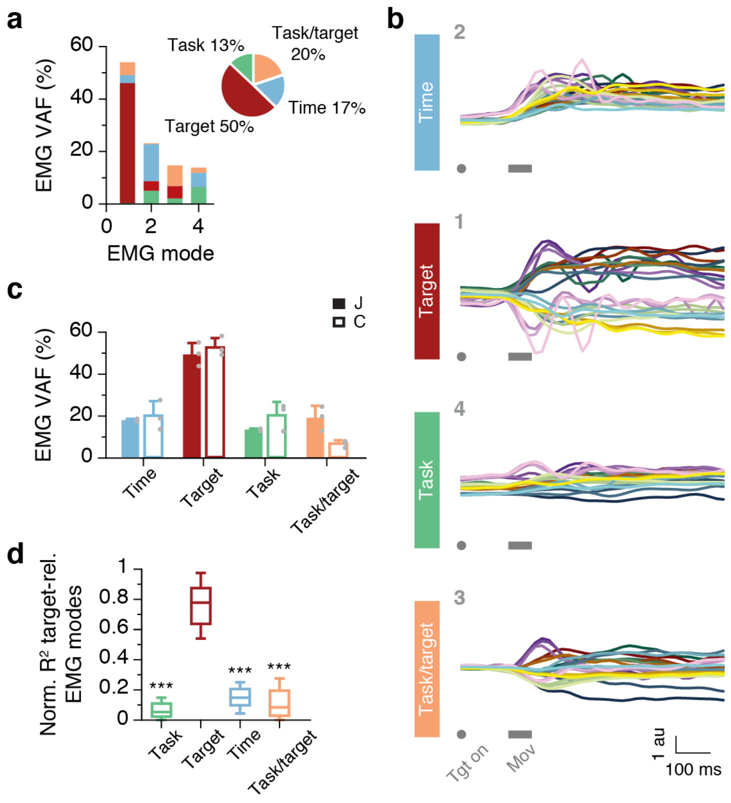

Supplementary Figure 8. Role of the target-related latent neural activity in the generation of EMGs. Demixed PCA (dPCA) of the covariation of the EMGs from all the tasks within a session: **(a)** Variance accounted for (VAF) by each EMG mode and its relation to behavioral parameters, for the example session from Monkey J shown in Fig. 4. EMG dPCs were more mixed in terms of behavioral covariates than the corresponding neural dPCs (see Fig. 4a). Inset: amount of VAF associated with each behavioral parameter across all four modes. **(b)** Activity of the four EMG modes, identified by behavioral parameter they are most strongly associated with. The number on the top left of each panel indicates the ranking of that EMG mode in terms of EMG VAF, as in (a). Each row corresponds to one behavioral parameter (vertical labels on the left). Each panel has 24 traces, corresponding to each of the 24 task/target combinations of wrist tasks; color code shown in Fig. 4d. **(c)** EMG VAF for each behavioral parameter, averaged over all the wrist tasks for all sessions from Monkeys J and C. Bars: mean + s.d; grey circles: individual sessions. Across all datasets, 51.3 ± 5.0 % of the total EMG variance was accounted for by EMG modes with target-related but task-independent activity. **(d)** Normalized R^2 of the predictions of the activity of target-related EMG modes obtained from four different decoders, each based on the two leading neural modes most strongly related to each of the four dPCA behavioral parameters. Performance was averaged over all muscles, tasks, and monkeys. Boxplots show median: center line; interquartile range: box; and data range (0.5 × interquartile range): whiskers. The *** denote P~0 (two-sided Wilcoxon rank sum test). As expected, target-related EMG modes were well predicted only by a decoder based on target-related neural modes (cross-validated normalized R^2: 75.6 ± 19.1 %).
